# Sourdough Breads Made with Selected Lactobacillus Strains and Spelt Flour Contain Peptides That Positively Impact Intestinal Barrier

**DOI:** 10.3390/foods14183184

**Published:** 2025-09-12

**Authors:** Costanza Cicchi, Manuela Leri, Monica Bucciantini, Viola Galli, Simona Guerrini, Ángela Jiménez-Ortas, Diego Ceacero-Heras, Olga Martínez-Augustín, Luigia Pazzagli, Simone Luti

**Affiliations:** 1 Department of Experimental and Clinical Biomedical Sciences, University of Florence, Viale Morgagni 50, 50134 Florence, Italy; costanza.cicchi@unifi.it (C.C.); manuela.leri@unifi.it (M.L.); monica.bucciantini@unifi.it (M.B.); simone.luti@unifi.it (S.L.); 2 Food Micro Team Ex-Academic Spin Off, Florence University, Via Santo Spirito 14, 50125 Florence, Italy; 3 Department of Agriculture, Food, Environment and Forestry (DAGRI), University of Florence, Piazzale delle Cascine, 18, 50144 Firenze, Italy; simona.guerrini@unifi.it; 4 School of Pharmacy, Ibs.Granada and CIBERehd, University of Granada, Campus de Cartuja s/n, 10871 Granada, Spain; ajimenezo@ugr.es (Á.J.-O.); dch@ugr.es (D.C.-H.); omartine@ugr.es (O.M.-A.)

**Keywords:** bioactive peptides, sourdough fermentation, antioxidant activity, anti-inflammatory activity

## Abstract

Cereal grains have been dietary staples for millennia, providing essential nutrients alongside their primary carbohydrate content. Recently, the search for sustainable, nutrient-rich alternatives has drawn attention to spelt (*Triticum aestivum* ssp. *spelta* L.), a low-input crop with promising nutritional properties. Spelt supplies a higher content of unsaturated fatty acids and minerals, such as iron, zinc, and magnesium and exhibits lower levels of phytic acid compared to common wheat. This study explores the nutraceutical potential of fermented bakery products made from spelt and wheat flours using sourdough fermentation, a process driven by lactic acid bacteria (LAB) and yeasts. Breads produced with baker’s yeast were included for comparison. Specifically, this manuscript focuses on the generation of bioactive peptides (BPs), which have demonstrated anti-oxidant, anti-inflammatory, and gut-protective effects by modulating oxidative stress and inflammatory signaling pathways. By comparing aqueous extracts from breads prepared with varying flours and fermentation methods, optimal conditions for producing functional baked goods could be defined. The findings may offer new avenues for developing bakery products that potentially increase intestinal health while promoting sustainable agriculture through the use of spelt.

## 1. Introduction

For thousands of years, staple grains like wheat, rice, and maize have formed the basis of human diets worldwide, supplying between 50% and 70% of the daily energy content needs of the global population [1]. Although carbohydrates can be considered their main component, cereals have a valuable nutritional profile, supplying proteins (even though poor in some essential amino acids, namely lysine and threonine), lipids including fatty acids such as palmitic and linoleic acid, vitamins, and a significant amount of phosphorus, potassium, calcium, and zinc. Moreover, they also provide soluble and insoluble fibers and other bioactive compounds, such as carotenoids, flavonoids, and a great assortment of phytochemicals that are currently associated with the beneficial effects of wholegrain cereals consumption [1,2,3]. In recent years, environmental changes, the spread of pathogens, and population growth have increased the need for new environmentally friendly crops that advance biodiversity and sustainability [4,5,6]. There is increasing interest in spelt (*Triticum aestivum* ssp. *spelta* L.), which has long been limited to animal feed. It is a low-input alternative crop with potential adaptation to harsh ecological conditions and disease resistance [7,8]. Indeed, Ruibal observed that the consumption of spelt compared to wheat may have some nutritional advantages, such as a higher unsaturated fatty acid/palmitic acid ratio, (mainly due to a greater content of oleic acid, and a higher concentration of iron, zinc, copper, magnesium, and phosphorus) and a lower phytic acid content, which improves the intestinal bioavailability of mineral [9,10]. As regards to protein content, spelt seems to have a higher protein content, usually varying from 10.8–16.1%, compared to wheat 9.3–13.3%, but the amount is highly variable and strongly dependent on the cultivation conditions [10,11]. Protein content and composition are related with baking quality; a high glutenin content and a relatively low gliadin/glutenin ratio are positively correlated with proper technological features. Generally, the percentage of gliadin from common wheat is lower compared to spelt, whereas the opposite is observed for glutenin content. More in detail the gliadin fraction of spelt is composed of a higher percentage of α gliadin, and the glutenin is characterized by lower ω and β-gliadin content and a lower percentage of low-molecular-weight glutenin subunits compared to wheat [11]. Although, a worse-protein-quality spelt wheat may represent a valuable alternative to common wheat for the production of food with healthier features, while having a lower environmental impact.

In baked products, the nutritional value of spelt flour may be further enhanced by sourdough fermentation, the most conventional and effective tool to improve shelf life and sensory quality of the food [12,13,14]. Sourdough is a flour/water mixture, in which lactic acid bacteria and yeasts have developed spontaneously or have been added as a starter culture. In this process, yeasts and lactic acid bacteria, together with cereal endogenous enzymes, contribute to the degradation of anti-nutritional factors such as phytic acid, improving gluten digestibility and determining the accumulation of low-molecular weight compounds such as bioactive peptides (BPs) [15].

Although bakery yeast leavening is the most used production method in industrial bakeries, the interest in sourdough fermentation is increasing because of the sensory properties, shelf-life, and improved nutritional value of the products. In fact, sourdough fermentation induces changes in metabolites, potentially having healthy properties and naturally leads, among other effects, to the release of bioactive peptides [16]. During sourdough fermentation, cereal proteins are digested both by endogenous cereal enzymes, activated by the low pH value of the medium, and by bacterial proteases, thus determining the liberation of various sized peptides which are more easily digested by intestinal proteases [17]. Reduction in the anti-nutritive factor phytic acid and the increase in organic acids and phenolic compounds, as well as the production of exopolysaccharides (EPS), contribute to improve nutritional qualities and bread softness, respectively [17]. Finally, sourdough fermentation reduces the level of FOODMAPs (Fermentable Oligosaccharides, disaccharides, monosaccharides, and polyoils) and decrease the glycemic index by starch retro-degradation and decrease in free glucose [18]. Ultimately, the peptidases from LABs liberate free amino acids which are then subjected to modification by the same micro-organisms [18].

The peptides released from proteins during fermentation manifest a broad spectrum of biological activities, such as antioxidant and anti-inflammatory activity [19,20,21]. Bioactive peptides have attracted a lot of attention due to their ability to affect the metabolic and physiological functions in the body. Most bioactive peptides (BPs) are encrypted in the structure of proteins and must be released through hydrolysis to acquire biological activity. The use of BPs for food preservation and fortification is usually regarded as safe, since no toxic or immunogenic effect has been detected for isolated peptides both in vitro and in vivo, even when they were administered at doses higher than those required to observe pharmacological activities [22]. Furthermore, BPs are biodegradable, do not accumulate in organisms, and are easily excreted, thereby preventing the adverse consequences usually associated with the toxicity of synthetic drugs [23,24].

Specifically, recent studies highlighted the ability of dietary BPs to reduce oxidative stress and general inflammation underlying many chronic diseases, mainly by scavenging reactive oxygen species (ROS) and regulating the KEAP1-NRF2 and the TLRs/MyD88/NF-κB pathway, together with iNOS inhibition [25,26,27,28,29]. Interestingly, some food-derived bioactive peptides have also been shown to improve intestinal barrier functionality and integrity by enhancing enterocytes differentiation, modulating the expression of tight-junction proteins such as Occludin, ZO-1, and Claudins, and inhibiting the over-activation of the MLCK pathway, responsible for cytoskeletal rearrangement in intestinal epithelial cells [30,31,32]. Both ex vivo and in vivo experiments have been performed to assess the effect of BPs on the intestinal epithelium, suggesting a theoretical basis for the use of BPs as dietary supplements [33,34,35,36]. In addition, there is emerging evidence that BPs-related nutraceutical properties of fermented products are actually retained after the baking process [37].

Under these premises, the fermented bakery product might be better tolerated by patients affected by chronic inflammatory conditions, such as Inflammatory Bowel Disease (IBD) [38,39]. Moreover, differences in protein content and composition between wheat and spelt suggest that the bioactive peptides formed during leavening may differ according to the type of flour.

The aim of this manuscript is to characterize aqueous extracts obtained from breads prepared with different flours (spelt and wheat) and leavening methods (sourdough and bakery yeast). Since previous results highlighted antioxidant and anti-inflammatory activities of peptides obtained from wheat flour fermented with selected lactobacilli strains [25,37], we used the same approach with spelt and wheat flour to identify the optimal conditions for the production of a fermented bakery product with improved nutraceutical properties.

## 2. Materials and Methods

### 2.1. Microorganisms Growth Conditions and Enumeration

Three lactic acid bacteria strains, *Companilactobacillus farciminis* (A11 and H3) and *Fructilactobacillus sanfranciscensis* (I4), previously characterized for their capacity to increase antioxidant and anti-inflammatory properties in sourdough and breads, were used [25,37]. The strains were regularly maintained for 24 h at 30 °C in MR3i medium [25]. *Saccharomyces cerevisiae* LV8 strain was aerobically cultured at 30 °C in MYPG (malt extract 5 g/L, yeast extract 3 g/L, meat extract 5 g/L and glucose 10 g/L). The strains were recovered from different Italian sourdoughs and belonged to the culture collection of DAGRI (Department of Agriculture, Food, Environment and Forestry of the University of Florence, Italy). Microorganism enumeration was performed by diluting and homogenizing 10 g of the dough samples with 90 mL of a sterile physiological solution. The LAB suspensions (100 µL) were plated on the MR3i medium using the pour plate method and counted after incubation for 48–72 h at 30 °C under anaerobic conditions. *S. cerevisiae* suspensions were plated on the MYPG agar and the colonies counted after incubation for 48 h at 30 °C under aerobic conditions. The counts were performed in duplicate.

### 2.2. Sourdoughs and Breads Preparation

For sourdough preparation, cultures of each LAB strain, grown overnight in the MR3i broth, were inoculated at a concentration of ca 10^8^ CFU/g, together with the *S. cerevisiae* strain LV8, at a concentration of ca 10^7^ CFU/g, into doughs with a dough yield (DY = dough weight × 100/flour weight) of 200. The sourdoughs were obtained by mixing water and commercial wheat (*Triticum aestivum* L.) flour (type ‘00’; COOP, Casalecchio di Reno, Bologna, Italy) (protein 12.5, fat 0.70, moisture 13.8 and carbohydrates 73.0 g/100 g) or spelt (*Triticum aestivum* ssp. *spelta* L.) flour (type ‘0’; COOP, Casalecchio di Reno, Bologna, Italy) (protein 14.0, fat 1.0 and carbohydrates 74.0 g/100 g) and then incubated at 30 °C for 18 h. Four breads were prepared (Table 1) as follows: two breads fermented by the prepared sourdoughs, either made by wheat (W-LAB) or spelt (S-LAB), and two breads fermented only by baker’s yeast (Zeus Iba, Florence, Italy), W-Sac, and S-Sac. The doughs with the sourdoughs were fermented for 4 h at 28 °C and then cooked in an oven for 40 min at 180 °C. The baker’s yeast doughs were fermented for 1 h at 28 °C and cooked for 40 min at 180 °C. Sourdough and dough fermentation were monitored by determining the pH (pH meter is from Metrohm Italiana Srl, Varese, Italy). Total titratable acidity (TTA) was determined using 10 g of dough that was homogenized with 90 mL of distilled water for 3 min. Results were expressed as the volume (mL) of 0.1 N NaOH required to reach a pH of 8.5. Then, each dough (200 g) was placed in a graduated cylinder (1 L) to assess the increase of dough volume during fermentation. The volume (in mL) was measured immediately and after fermentation at 30 °C before baking. The leavening was calculated by: [(Vfin − V0)/V0] × 100, (Vfin was the final volume and V0 was the initial volume).

### 2.3. Extraction, Characterization, and Fractionation of Water-Soluble Extracts

At the end of fermentation, the water-soluble extracts (WSEs) were obtained by extracting dried breads and sourdoughs with sterile water (1:3 *w/v*) and then centrifuged at 14,000× *g* for 20 min at 4 °C according to Galli [25].

#### 2.3.1. Free Amino Acid Determination in Water–Salt-Soluble Extracts

For the free amino acids (FAAs) determination of WSE the breads, samples were diluted with distilled water (1:10 *w/v*) and then filtered with Amicon^®^ Ultra-4 Centrifugal Filters (3000 Da, Merck Millipore, Burlington, MA, USA) before injection. FAA of WSE were obtained by High-Performance Liquid Chromatography (HPLC) analysis using an Agilent 1260 Infinity II system (Agilent Technologies Inc., Santa Clara, CA, USA) consisting of a quaternary pump, a vial sampler, a column heater, a fluorescence detector, a diode array detector WR, and a multicolumn thermostat. Separation was obtained with a Kinetex 5 µm C18 100 Å column (150 × 4.60 mm; Phenomenex, Castel Maggiore, Bologna, Italy) under the following conditions: mobile phases pH 6.5 buffer acetate/methanol 95:5 (solvent A) and methanol/acetonitrile 70:30 (solvent B); elution was carried out at 40 °C with flow rate 1.0 mL/min as reported in Kelly [40]. The reaction mixture was made according to the manufactures instruction and contained: 1 µL of sample extracts, 0.5 µL of o-phthaldialdehyde (OPA) and 2-mercaptoetanol solution (derivatization agent), 2.5 µL of 0.2 M Na_2_B_4_O_7_ × 10H_2_O (pH 9.5) solution, 0.4 µL of 9-fluorenylmethyl chloroformate (FMOC), and 32 µL of solvent A and 0.4% of H_3_PO_4_. Data were collected and analyzed using the OpenLab CDS Workstation (Agilent Technologies Inc., Santa Clara, CA, USA). Quantitative analysis was carried out by standard curves designed for each compound.

#### 2.3.2. Isolation of Low Molecular Weight Peptides

Peptides were fractionated from the WSEs according to the method previously described in Luti [37]. Firstly, WSEs were assayed for protein and peptide content by the BCA method [41]. Each sample (5 mg/mL) was added to 0.05% (*v/v*) trifluoroacetic acid (TCA) and then centrifuged at 10,000× *g* for 10 min to remove impurities. Reverse Phase HPLC (Ultimate3000, ThermoScientific, Waltham, MA, USA) equipped with a C18 column (Kinetex, 4.6 250 mm, 5 m, 100 Å, Phenomenex, Torrance, CA, USA) and a UV detector operating at 214 nm were used to analyze the samples. Elution was carried out in water/acetonitrile gradient in the presence of 10 mM trifluoroacetic acid (TFA) at 0.8 mL/min flow. Eluted peptides were manually collected from 7 to 15 min retention time. Solvents were removed from samples via freeze-drying and peptides were re-dissolved in PBS. Peptide content was detected by the Bradford method [42].

#### 2.3.3. Mass Spectrometry Analysis

A solution containing approximately 20 µg of peptide was added with 100 μL of 1 mM DTT in 0.1 M NH_4_HCO_3_ and the mixture was kept in an orbital shaker at 56 °C for 45 min and finally removed. Alkylation was performed using 100 μL of 55 mM iodoacetamide in 0.1 M NH_4_HCO_3_: 20 µg of peptide previously reduced in 10 mM DTT were added and the solution was shaken for 30 min at room temperature in the dark. The iodoacetamide solution was then removed, and 100 μL of 0.1 M NH_4_HCO_3_ were added. After shaking for 10 min, the solution was discarded. Subsequently, 50 μL of 0.5% acetic acid in H_2_O:actetonitrile (20:80, *v/v*) was added. The solution was concentrated, the final volume was adjusted to 20 μL with 0.5% acetic acid, and finally it was applied to nano liquid chromatography (nLC) coupled to high-resolution mass spectrometry (HRMS) equipped with a nanoelectrospray (nESI) interface (nLC-nESI-HRMS/MS). The instrument was composed of an EASY-nLC 1200 connected to LTQ Orbitrap hybrid mass spectrometer (Thermo Scientific, Bremen, Germany). The instrument was set as follows: nESI spray potential 1.8 kV, capillary and tube lens voltages 42 and 120 V, respectively. Acclaim PepMap 100 C18, 3 μm, 100 Å, 75 μm × 150 mm (Thermo Scientific), operating at 0.3 μL/min flow rate was used as column. Solvent A was 100% water and solvent B was 80% acetonitrile/20% water, both containing 0.1% formic acid; (Sigma, Rome, Italy). The nLC-nESI HRMS/MS system was injected with 1 μL. Elution was done by linear gradient starting from 2% B for 5 min, to 40% B in 340 min, to 90% B in 5 min.

MS data acquisition was made in data-dependent acquisition mode, with a HRMS full scan from 300 to 1600 m/z in the Orbitrap analyzer, using a 1 × 10^6^ target value. MS/MS spectra were acquired in the linear quadrupole ion trap analyzer: precursor ions were selected from the 7 most intense signals in the HRMS full scan spectrum above 600 a.u. threshold and an isolation window of 2.2 Da. Normalized collision energy of 35% and 20 ms activation time were used. The acquired data were analyzed using the Mascot 2.4 search engine (Matrix Science Ltd., London, UK) against a Triticum and a Spelta database created from NCBI. Searches were performed allowing the following: (i) no enzyme, (ii) carbamidomethylation of cysteine as fixed modification, and (iii) oxidation of methionine and acetylation and amidation as variable modifications.

### 2.4. Biological Activity of Peptides on RAW 264.7 Cells

Murine macrophages were used to evaluate the anti-oxidant activity of peptides [43,44]. RAW 264.7 cells (LGC limited, Augsburg, Germany) were cultured in standard conditions under humidified atmosphere (5% CO_2_, 37 °C) using Dulbecco’s Modified Eagle Medium (DMEM) supplemented with 10% (*v/v*) fetal bovine serum (FBS), 1 mM glutamine and 100 μg/mL penicillin/streptomycin. Cells were passed twice a week and culture medium was refreshed every 2 days. For each experiment, cells that were not subjected to any treatment served as the control group

#### 2.4.1. Cell Viability

The impact of peptides on cell viability was determined by treating cultured RAW 264.7 cells. The cells were seeded in fresh medium (DMEM) on a 24-well plate at a density of 300,000 cells/mL. Peptides were added to each well at a final concentration of 0.05 mg/mL. After 24 h of incubation, cell viability was measured using the 3-(4,5-dimethylthiazol-2-yl)-2,5-diphenyltetrazolium bromide (MTT, Sigma-Aldrich, St Louis, MO, USA) method, which is based on the ability of succinate dehydrogenase to convert MTT to visible formazan crystals in viable cells. For each well, 400 μL of MTT solution was used. After 30 min incubation, the MTT solution was thrown away and the formazan salts were solubilized in pure DMSO. The absorbance for each well was determined at 595 nm in a microplate reader (Biotek Synergy H1 Plater Reader, Agilent, Santa Clara, CA, USA). Cell viability was reported as a percentage of the control absorbance.

#### 2.4.2. Reactive Oxygen Species (ROS) Assay

RAW 264.7 cells, were cultured in 24-well plates at a density of 300,000 cells/mL. Cells were incubated in complete medium for 24 h after plating. Starvation medium was added to the cells 6 h prior treatment. Peptides were added to 0.05 mg/mL final concentration., LPS was added to a final concentration of 1 μg/mL to induce ROS production and kept 1 h in incubation., Intracellular ROS were measured after 24 h co-treatment using the oxidation of the fluorescent probe H2DCF-DA (2,7-dichlorodihydrofluorescein diacetate) 10 μM in DMSO according to Luti [37]. The probe was added to the medium, and cells were incubated for 1 h. The ROS production was measured using a Biotek Synergy H1 Plater Reader (Agilent, Santa Clara, CA, USA). The fluorescence signal (fluorescence intensity units) was normalized to the protein content determined by the BCA assay. The results were presented as a percentage of reduction in ROS formation.

### 2.5. Biological Activity of Peptides on Caco-2 Monolayer

Caco-2 cells (human colorectal adenocarcinoma) were purchased from Sigma (Italy). Cells were cultured under standard conditions as reported for RAW cells. They were routinely passaged once a week and 0.5 mL of culture medium was refreshed every 2 days until confluence. According to the literature, they were used to evaluate the effect of peptides on intestinal barrier integrity and functionality [45,46].

#### 2.5.1. Intestinal Epithelium Differentiation and Cell Viability

To assess successful monolayer differentiation, an alkaline phosphatase assay was performed according to Cicchi [30]. Briefly, Caco-2 cells were grown in 24-well plates for 21 days, with medium changes every 2 days. After formation of the epithelial monolayer, alkaline phosphatase activity was assessed by adding 0.5 mL of 2.5 mg/mL pNPP (*p*-nitro-phenyl phosphate) solution in 100 mM Glycine; 2 mM MgCl_2_ (pH 9.8). After incubating at 37 °C for 20 min, the formation of yellow color was assessed to confirm enterocytes differentiation.

For cell viability assessment, peptides were added to the differentiated monolayer at a final concentration of 0.1 mg/mL. The MTT assay was executed as described in Section 2.5.1. Untreated cells were used as controls.

#### 2.5.2. Intestinal Barrier Permeability

To check the effect of the peptides on intestinal permeability and functionality, we examined the transport of 10 kDa FITC-Dextran across the intestinal epithelial monolayer. Caco-2 were seeded at 60,000 cells/mL density on Transwells in 24-well plates. The cells were grown to confluence and differentiation (approximately 21 days post plating), with medium refresh every 2 days. Differentiated monolayers were then treated with peptides at a final concentration of 0.1 μg/μL for 2 h. Then, LPS 20 μg/mL was added to each well (with the exception of the control) and after 24 h incubation, FITC-Dextran was added to a final concentration of 100 μM. Fluorescence was read every 15 min for 2 h (λex 485 nm and λem 535).

#### 2.5.3. Monolayer Immunofluorescence Staining

Caco-2 cells were plated on sterilized glass coverslip in 24-well plates for 21 days, and the medium was changed every 2 days. Treatment with peptides and LPS was performed as described in 2.7.4. After treatment, the medium was discarded and cell nuclei were stained with Hoechst 33,342 diluted in PBS at a concentration of 5 μM for 30 min. The cells were then fixed in 2% paraformaldehyde for 6 min at 37 °C and washed twice in PBS. After blocking in BSA 2% and gelatin 2% for 30 min, monolayers were incubated with primary antibody anti-CX43 (1:500, rabbit) for 1 h. Secondary anti-rabbit antibody conjugated to Alexa 568 (1:100) was added to each wall and incubated for 1 h at room temperature. The cells were washed twice in PBS and once in deionized water. Multicolor images were captured using a scanning microscope (Leica TCS SP8, Leica Microsystem srl, Buccinasco, Italy) equipped with a 63× HCX Plan APO lens with an NA of 1.4–0.6 and an oil immersion objective. Images were captured with Leica LAS-AF imaging software (Versions 5.1.0). and analyzed using FiJi software (Version 9.3.1).

### 2.6. Inflammatory and Immune Activation Markers in Mouse Jejunum Organoids

Jejunal organoids were explanted and cultured according to Cordova [47]. Jejunum intestinal organoids were dissected and incubated for 30 min at 4 °C in PBS with 2 mM EDTA from wild-type (WT) mice. After shaking, dissociated fragments were filtered through 70 µm filter and crypts were counted and centrifuged for seeding.

The pellet was resuspended in Corning-Matrigel^®^ (Fisher Scientific, Madrid, Spain) and IntestiCult^TM^ (StemCell, Grenoble, France) with a 1:1 ratio. The domes were cultured in 24 well plates with IntestiCult^TM^ that was supplemented with penicillin–streptomycin (Sigma-Aldrich). For RNA expression analysis, total RNA was obtained using the RNeasy Mini Kit (Qiagen, Barcelona, Spain). One µg was retro-transcribed, and specific RNA sequences (Table S1) were amplified with a Bio-Rad CFX Connect real-time PCR device (Bio-Rad Hercules, CA, USA). The 2−ΔΔCT method was used for relative quantification using 18 s, Hprt (Hypoxantine phosphoribosyltransferase) and Ppib (peptidylprolyl isomerase B) as reference genes. Control organoids consisted of those not treated with peptides.

### 2.7. Statistical Analysis

Data are presented mean ± standard deviation (SD) from at least three independent experiments. Statistical analysis was performed using t-Student or one-way ANOVA for normally distributed data sets. Tests were performed in GraphPad Prism 5, setting statistical significance at *p* < 0.05.

## 3. Results

### 3.1. Dough Analysis

Three selected bacterial strains, namely L. *Companilactobacillus farciminis* (A11 and H3) and *Fructilactobacillus sanfranciscensis* (I4), were inoculated in sourdoughs prepared with wheat and spelt flour together with *S. cerevisiae* LV8. Sourdoughs were fermented for 18 h at 30 °C. No differences based on the type of flour were observed and the final pH of the sourdoughs was 3.92 and 3.98, for wheat and spelt sourdoughs, respectively. LAB reached ca 9 log CFU/g, while *S. cerevisiae* attained to ca 7 log CFU/g, in both the sourdoughs. As regards bread-making, Table 2 shows the main chemical and microbiological features of experimental doughs.

The acidification parameters (pH and TTA) of the bread before cooking were in line with those reported for sourdough breads, showing a pH < 4.5 and a TTA higher than 3.5 mL, thus indicating a proper activity of all the LAB strains. The microorganism concentration results are consistent with the values generally observed in sourdough, where the LAB:YS ratio varies from 100:1 to 10:1 [48]. The dough volume increase was not different among the samples, particularly in the sourdough breads compared to dough leavened with baker’s yeast, despite the higher yeast concentrations found in the latter. As for the sourdoughs, the type of flour did not affect dough features.

### 3.2. Extraction and Characterization of Water-Soluble Extracts

Protein content of WSE from each sample is reported in Table 3. Protein content in each sample is significantly different, in particular comparing samples fermented with *S. cerevisiae* and selected LAB starters (W-Sac vs. W-LAB and S-Sac vs. S-LAB, *p* < 0.001). No significant difference in protein content was highlighted between samples obtained with different flours but subjected to the same type of fermentation (W-Sac vs. S-Sac and W-Sac vs. W-LAB, *p* > 0.2).

#### 3.2.1. Free Amino Acid Content in the WSE Extracts

The free amino acid content of the extracts was not statistically different among W-LAB, S-Sac, and S-LAB samples, while the W-Sac showed a lower content (Table 4).

**Figure 1:**
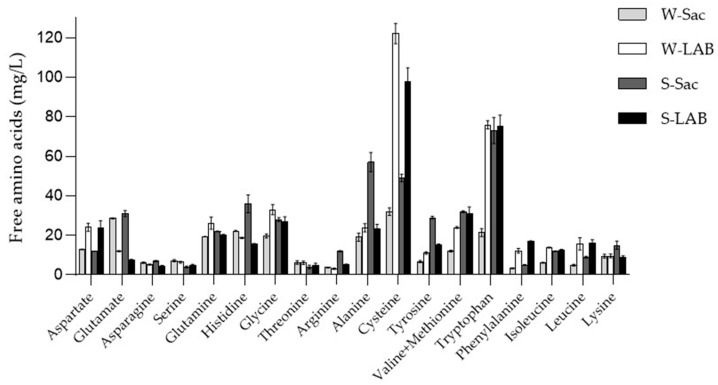
Amino acid concentration of the extracts. Amino acids were detected by OPA derivatization and quantified by a standard solution. Results are expressed as average ± standard deviation. The detection was performed by a fluorescence detector fluorescence detector set at 345 nm (λex) and 455 nm (λem) and UV detector (λ = 338 nm).

No statistically significant differences in the FAA content were detected between samples obtained with different flours but subjected to sourdough fermentation. In wheat samples, fermentation by lactobacilli increased the FAA content compared to bread with baker’s yeast, in agreement with Gänzle [16]. On the contrary, the spelt sample FAA was not different based on the type of fermentation (Figure 1). The most abundant amino acid found in the WSE sourdough was cysteine, representing 27% and 24% of the total amino acid in W-LAB and S-LAB, respectively, followed by tryptophan. Cysteine was found to be the most abundant amino acid also in the W-Sac WSE, whereas tryptophan was the most abundant in the S-Sac WSE.

#### 3.2.2. Fractionation of the Peptides

**Figure 2:**
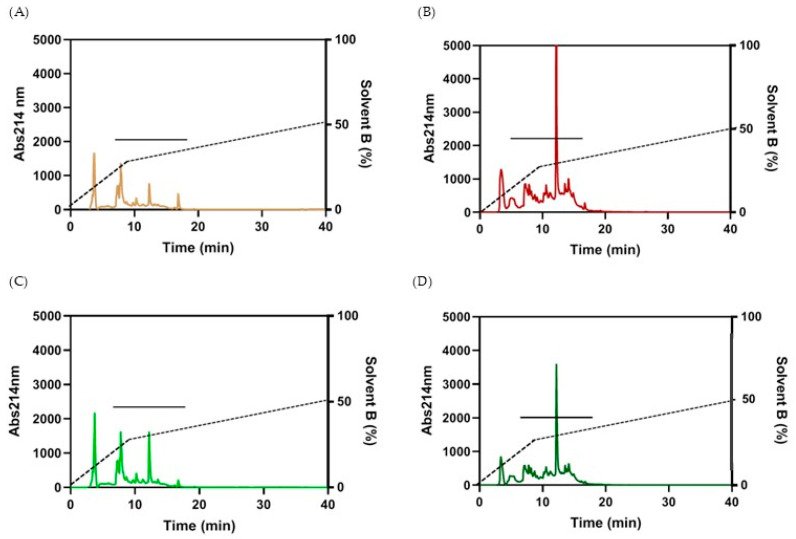
RP-HPLC of water extracts for peptide fractionation. 500 µL aliquot was applied on a Reverse Phase and eluted with a non-linear gradient (Solvent A: H_2_O + 10 mM TFA; Solvent B: CH_3_CN + 10 mM TFA. (-----): Gradient: 0–10 min 30%B, 10–50 min 50%B, 50–60 min 100%B). (^_____^): collected peptides. Peptides from: (**A**): W-Sac: dough with wheat flour fermented by baker’s yeasts; (**B**): W-LAB: dough with wheat flour fermented by a sourdough; (**C**): S-Sac: dough with spelt flour fermented by baker’s yeasts; (**D**): S-LAB: dough with spelt flour fermented by a sourdough.

RP-HPLC was used to fractionate the peptide/protein content and to remove any free amino acids, sugars, and other soluble metabolites occurring in the aqueous solvent used for extraction. About 500 μL peptides were then injected in the RP-HPLC for each run. As shown in Figure 2, peptides from wheat and spelt flour showed similar chromatographic profiles. The major differences in the elution of peaks depended on the type of fermentation. In particular, flours fermented with selected LAB starters exhibited a higher amount of peptides, in agreement with protein quantification. A higher peak at around 12 min elution time is common to both W-LAB and S-LAB. The eluted fractions were collected, freeze-dried, and then re-suspended in cell-cultured medium at 1 mg/mL concentration to evaluate their biological activity in cultured cells.

#### 3.2.3. Peptide Content Analysis by Mass Spectrometry

Peptides obtained from HPLC fractionation were subjected to mass spectrometry to identify those derived from the leavening process and highlight any differences between the samples. The analysis also allowed the identification of the proteins from which the various peptides derive by a target decoy search. Database entries were identified after excluding protein contaminants, such as keratins, retaining only proteins with at least one unique peptide per protein. The proteins matching the peptides mainly belong to prolamins, typical cereals nonpolar proteins rich in leucine, proline, and glutamine. Other sources of peptides are gliadins, glutenins, and inhibitors of α-amylases (Table S2). In all the analyzed samples Gln, Val, and Pro and Gly are the most represented amino acids, while unique peptides in spelt (both Sac and LAB) appear to contain a generally higher His content (Figure S1). However, the analysis of the amino acid composition of each sample does not highlight relevant differences in amino acids content: essential hydrophobic amino acids (Ile, Leu and Val) account for 20.9%, 22.7%, 19,9%, and 21,9% of the total in W-Sac, W-LAB, S-Sac, and S-LAB, respectively (Table 5). Conversely, Lys is present in a low percentage in peptides from bakery’s yeast (1.9%) and increase in peptides from sourdough fermentation (3.9% and 2.8% in W-LAB and S-LAB, respectively (Table 5).

**Figure 3:**
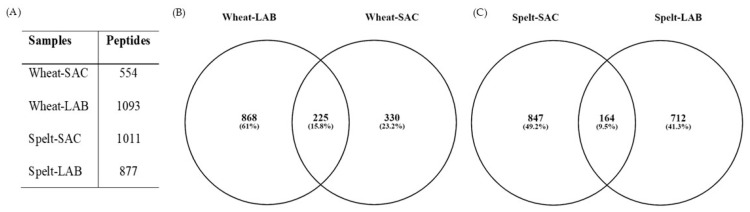
Peptide identification and overlap in *Triticum* and *Spelta* samples. (**A**) Number of peptides identified in each sample using the Mascot 2.4 search engine against a Triticum and a Spelta database created on 24 July 2024. (**B**,**C**) Venn’s diagrams obtained at https://bioinfogp.cnb.csic.es/tools/venny/index.html [49] (accessed on 10 June 2025). Numbers refer to the number of peptides typical of each sample and to the peptides sharing common sequences.

The number of identified peptides is different both based on the flour used and the leavening method (Figure 3A). The different number of peptides found in the samples obtained with wheat flour can be attributed to the greater richness of the database relating to *Triticum aestivum* compared to *T. spelta* and only an intra-specific comparison can be made. The sourdough process led to a marked increase in the number of peptides in the wheat flour samples, consistent with previously reported results (Figure 3A) [25,37]. The number of peptides almost doubled (W-Sac vs. W-LAB samples) and only the 13% overlap between samples obtained with baker’s yeast and those obtained with sourdough fermentation (Figure 3B). On the contrary, the use of Lactobacilli does not significantly influence the number of peptides from spelt flour; in fact, the S-Sac extract presents peptides even slightly larger than those found in the S-LAB sample (Figure 3A). Notably, only 8.7% of the peptides overlapped between S-Sac and S-LAB (Figure 3C), highlighting differences in bioactive peptides generation depending on the food matrix used. Moreover, qualitative differences in the peptide sequences obtained from the extracts are evident: only sixty-four peptides are common to all the samples and only eight peptides are common between W-LAB and S-LAB, thus confirming the importance of both acid fermentation and food matrix in the production of peculiar peptide sequences (Figure S1).

### 3.3. Effect of Peptides on RAW 264.7 Cell Viability and ROS Production

**Figure 4:**
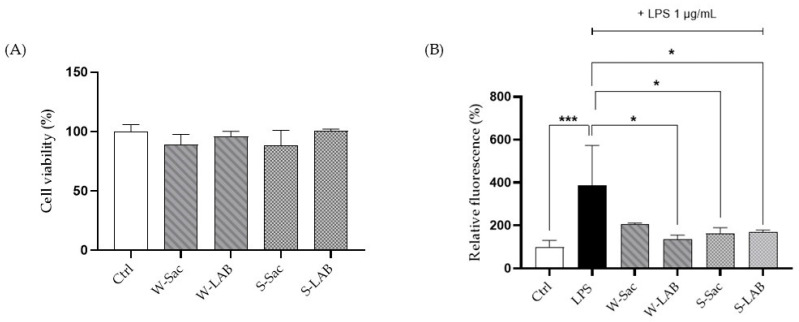
Effect of peptides on cell viability and ROS production. (**A**) Determination of cell viability. RAW 264.7 cells were treated with peptides (0.05 mg/mL) for 24 h, after which formazan absorbance was measured at 595 nm and cell viability was quantified as percentage. (**B**) Intracellular ROS levels in RAW cells. Ctrl: control, untreated cells; LPS: cells treated with 1 μg/mL LPS, positive control. W-Sac, W-LAB, S-Sac, and S-LAB: cells incubated with 0.05 mg/mL peptides and after 1 h, with 1 µg/mL LPS. Results are expressed as the mean ± SD of three experiments performed in triplicate. Statistical analysis was done for each sample vs. the LPS value (* *p* < 0.05; *** *p* < 0.001).

Cell viability of macrophages treated with the peptides was assessed using the MTT assay to ensure that they did not induce toxic effects on the cells (Figure 4A). The results showed that cell viability was not affected, as values from treated RAW 264.7 cells did not differ significantly from those of the respective controls.

The evaluation of a possible healthy effect of the peptides was assessed on RAW cells, previously stressed with LPS using the fluorescent probe DCFH-DA. Given the ability of LPS to activate inflammatory pathways and enhance reactive oxygen species production, LPS-treated cells were used as positive controls. Each of the peptide samples is capable of reversing the free radical formation induced by LPS itself: W-LAB, S-Sac, and S-LAB peptides provided a strong effect on RAW cells showing an antioxidant activity capable of restoring the ROS values to the levels observed in control cells. Instead, W-Sac peptides reduced ROS to a lesser extent, not statistically different from the ROS level of the positive control (Figure 4B).

### 3.4. Impact of Peptides on Intestinal Permeability and Barrier Integrity

To assess the effects of peptides on intestinal permeability and barrier integrity, Caco-2 monolayers cultured in Transwells were used as an in vitro model of the intestinal epithelium. In particular, Caco-2 is a human colorectal adenocarcinoma cell line that differentiates into functional enterocytes after reaching confluence expressing typical enterocyte markers such as alkaline phosphatase and tight-junction proteins.

#### 3.4.1. Cell Viability and Intestinal Barrier Permeability

**Figure 5:**
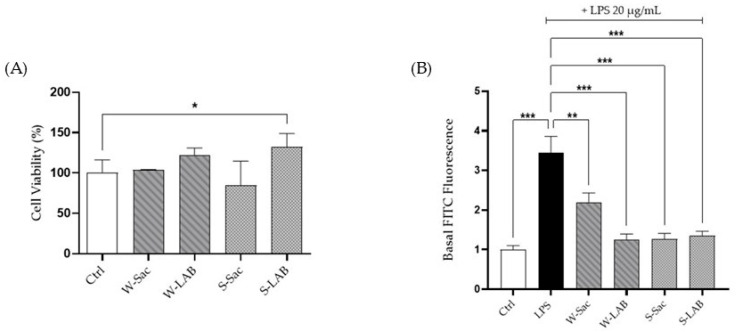
Impact of peptides on Caco-2 monolayer. (**A**) Effect on cell viability. (**B**) Effect on intestinal epithelia integrity. Ctrl: control, untreated cells; LPS: cells treated with 20 μg/mL LPS, positive control; W-Sac, W-LAB, S-Sac, and S-LAB: cells pre-treated for 2 h with 0.1 μg/μL peptides and then with 20 μg/mL LPS. Results are the mean ± SD of two different experiments performed in triplicate. Statistical analysis was performed for each sample with respect to the LPS treatment, as well as for the comparison between the LPS vs. control (* *p* < 0.05; ** *p* < 0.01; *** *p* < 0.001).

The effect of various peptides on the intestinal barrier was assessed using a Caco-2 monolayer cultured in a Transwell system for 21 days. Monolayer differentiation was confirmed through an alkaline phosphatase assay as detailed in Section 2.5.2. The potential cytotoxic effects of the peptides on the cell monolayer were assessed using the MTT assay. Peptides treatment did not impair cell viability (Figure 5A), and interestingly, the S-LAB treatment really showed a slight increase in cell viability. Intestinal permeability in response to peptide treatment was evaluated using the FITC-Dextran assay. As shown in Figure 5B, all peptide treatments significantly mitigated the barrier disruption caused by LPS, supporting their role in restoring epithelial integrity. In line with previously observed antioxidant properties, peptides derived from W-LAB, S-Sac, and S-LAB exhibited the most pronounced protective effects, demonstrating a stronger capacity to reinforce barrier function than W-Sac. Statistically significant differences were observed, with W-LAB, S-Sac, and S-LAB showing highly significant effects (*** *p* < 0.001), while W-Sac demonstrated a moderate but still significant improvement (** *p* < 0.01).

#### 3.4.2. Effects of Peptides on Gap-Junction Protein Expression

**Figure 6:**
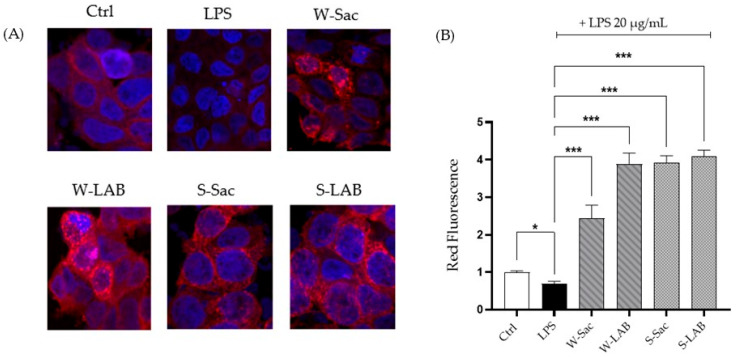
Expression of the CX43 gap-junction protein. Ctrl: control, untreated control cells; LPS: cells treated with 20 μg/mL LPS, positive control; W-Sac, W-LAB, S-Sac, and S-LAB: cells pre-treated for 2 h with 0.1 μg/μL peptides and then with 20 μg/mL LPS. (**A**) Semi-quantitative analysis of the red fluorescence compared to the control cells. Data are presented as a percentage relative to untreated cells. Results are the mean ± SD of three different experiments performed in triplicate. Statistical analysis was executed for each sample compared to value of LPS (* *p*< 0.05; *** *p* < 0.001 vs. LPS) and for LPS vs. control. A total of 300–350 cells were analyzed per condition. (**B**) Representative confocal microscopy images showing Caco-2 monolayer. Nuclei were labelled with the vital probe Hoechst 33,342 (blue signal); gap-junction protein was labelled with anti-CX43 antibodies, and anti-rabbit secondary antibodies (red signal) were conjugated with Alexa 568.

The positive impact of the peptides on the intestinal barrier function was further confirmed by confocal microscopy analysis of connexin-43 (CX43), a key gap-junction protein. LPS-induced epithelial injury resulted in a significant decrease in CX43 expression, as reflected in a reduced fluorescence signal (Figure 6A,B). However, treatment with specific peptides effectively reversed this downregulation, significantly enhancing CX43 expression compared to the control (untreated) condition (Figure 6A,B). This is evident from the intensified fluorescence signals observed in the confocal images, which highlight the peptides’ role in promoting epithelial repair and barrier recovery. Notably, peptides derived from W-LAB, S-Sac, and S-LAB demonstrated the most pronounced effects, whereas W-Sac-derived peptides did not elicit a significant response. This underscores the variable efficacy depending on the origin of the peptide.

### 3.5. Inflammatory and Immune Activation Markers in Mouse Jejunum Organoids

**Figure 7:**
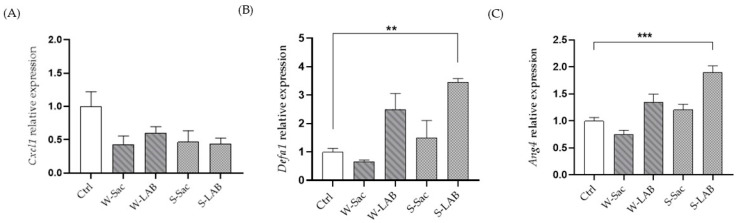
Peptide-Induced Modulation of Inflammatory and Antimicrobial Gene Expression in Mouse Jejunum Organoids. The effects of peptides on the inflammation marker Cxcl1 (**A**) and the antimicrobial peptides Defa1 and Ang4 (**B**,**C**) were investigated. Peptides were tested on mouse jejunum organoids at a final concentration of 0.05 mg/mL and for a 24 h treatment. Gene expression was quantified by RT-qPCR and expressed as relative expression compared to control organoids. Ctrl: control, untreated organoids, Results are the mean ± SD of two different experiments performed in triplicate (** *p* < 0.01, *** *p* < 0.001).

Gene expression analysis by RT-qPCR was used to investigate the role of peptides on inflammation and immune activation. We chose mouse jejunum organoids as they represent an intestinal model composed of heterotypic cell lineages typical of the original tissue. Treatment of jejunum organoids with peptides at a final concentration of 0.05 mg/mL determined some effects on inflammation and intestinal immune activation. First, peptides from all samples showed a decreasing trend in the expression of the inflammatory marker gene *Cxcl1* (Figure 7A). Even though not statistically significant, peptides from S-LAB have the strongest effect on *Cxcl1* reduction, with *p* = 0.0512.

Interestingly, peptides from samples fermented with selected LAB starters (W-LAB and S-LAB) affect the expression of the *Defa1* and *Ang4* genes, which encode two antimicrobial peptides. While the effect of W-LAB on the expression of these genes is not statistically significant, peptides from S-LAB cause a significant increase in the expression of both *Defa1* and *Ang4* (Figure 7B,C).

## 4. Discussion

Bread is one of the main foods of the Mediterranean diet and an extraordinary source of bioactive peptides, especially when obtained using sourdough fermentation. This traditional method ensures optimal sensory and shelf-life features, also improving the bread’s nutritional value, as demonstrated by recent clinical trials [50,51]. Some of the health-promoting properties of sourdough baked goods arise from the production of bioactive peptides, whose biological activities are preserved even after baking [37]. In addition to reassessing traditional bread-making methods, there has been a re-evaluation of ancient grains such as spelt, both for their nutritional characteristics and their ability to adapt to new climatic conditions related to global warming [52,53].

Based on these premises, in this manuscript we aimed at making a comparison between breads obtained with different types of flour (wheat and spelt) and different leavening methods (baker’s yeast and sourdough).

Firstly, the four samples (W-Sac: dough with wheat flour fermented by baker’s yeasts; W-LAB: dough with wheat flour fermented by a sourdough; S-Sac: dough with spelt flour fermented by baker’s yeasts; S-LAB: dough with spelt flour fermented by a sourdough) were characterized by their total protein and free amino acids content which was recovered in the aqueous extracts obtained from the respective breads. In particular, we found that sourdough fermentation is associated with a higher peptide content. Therefore, it is conceivable to hypothesize that both the specific protease activity and the acidification occurring during the fermentation process by LAB may be responsible for releasing unique peptide sequences as already reported in the literature [37,54]. Moreover, a higher peptide content was detected in the S-LAB extract, likely reflecting the greater protein content of spelt compared to wheat [17]. Considering these results, the content of free amino acids is lower in the W-Sac extract; conversely, the extracts from spelt flours, regardless of the fermentation technique, have a significantly higher quantity of amino acids. However, it should be considered that the protein and free amino acid content of the extracts cannot be directly compared to that of wheat and spelt flours [55], as most insoluble proteins are removed during the precipitation step.

The levels of individual amino acids are generally increased during sourdough fermentation and influenced by pH and leavening agent [56]. Among amino acids, a particularly high content of cysteine in the sourdough WSE was observed. Cysteine residues are contained in α- and γ-gliadins and are responsible for the reduction of disulfide bonds, thereby weakening the gluten network [57]. Cysteine is generally produced by cysteine proteinases, which are the most active protease group in wheat at around pH 4, which correspond to the pH of the W-LAB and S-LAB doughs [57].

In agreement with the protein content, the chromatographic fractionation of peptides from the aqueous extract shows different chromatographic profiles of samples fermented with LAB, compared to those obtained by bakery yeast fermentation. This observation was only partially supported by the mass spectrometry analysis, which revealed that sourdough fermentation increases the number of unique peptides in wheat flour compared with baker’s yeast fermentation. On the contrary, the number of peptides released from spelt flour remains consistent regardless of the type of leavening method. This may indicate a lower variability of the protein sequences present in wheat compared to spelt, resulting in a lower number of peptide species even if they are present in greater quantities as indicated by protein assays. Alternatively, the amino acid sequences of spelt proteins are more readily hydrolysable than those of wheat proteins, or, the endogenous proteases in spelt are most active despite the absence of a high acidic environment induced by LAB fermentation. In agreement with the literature [37], peptides from all samples are rich in glutamine, which prolamines of wheat and spelt are rich in. Unique peptides from spelt show a larger amount of His, but the overall analysis of the amino acidic composition does not allow us to highlight any significant difference among the samples.

The antioxidant activity of food-derived peptides has already been demonstrated in various food sources [58], along with their beneficial effects on intestinal epithelial integrity [59]. To the best of our knowledge, this study is the first to isolate peptides from spelt flour and to assess their biological activity using cellular models, including murine RAW 264.7 macrophages and Caco-2 intestinal monolayer. After confirming that peptides did not exhibit any toxic effects on either macrophages or intestinal cells, we evaluated their antioxidant activity which may be the basis of other beneficial activities such as anti-inflammatory, antimicrobial, and even anticancer effects [58]. In agreement with previous findings [37], peptides from W-Sac and W-LAB were able to prevent the ROS increase induced by LPS, with W-LAB peptides showing a higher antioxidant activity, even if not statistically significant. The same healthy properties were observed for peptides from spelt flour; however, in this case the peptides showed a similar, highly significant, reduction in antioxidant activity, regardless of whether they originated from breads obtained with bakery yeast or sourdough.

Among the many biological activities described in the literature [22,23,24], we focused on assessing the anti-inflammatory potential of the peptides in small intestine cells, as these represent the first site of interaction upon dietary intake. The Caco-2 (Cancer coli-2) cell model is widely recognized as a reliable in vitro surrogate for studying human intestinal absorption [46]. Peptides obtained from fermentation with LAB show a stronger effect on restoring epithelial barrier integrity after damage and induce better recovery of gap-junction protein Connexin-43 expression. These activities are present in all the analyzed samples; however, even if not statistically significant, W-SAC peptides increase intestinal barrier health, although less pronounced than that observed with the W-LAB, S-Sac, and S-LAB peptides, thus confirming the overall higher biological activity of peptides derived from spelt proteins regardless to the leavening method.

Experiments conducted on murine intestinal organoids further confirmed the enhanced biological activity of peptides derived from spelt fermented through the sourdough process. More specifically, spelt-derived peptides were found to impact intestinal inflammation by lowering the expression of the pro-inflammatory cytokine Cxcl1, which is usually upregulated during a wide range of inflammatory processes [60]. These peptides may also have an impact on intestinal immune activation, as they induce an increase in the expression of the antimicrobial peptides Defensin A1 and Angenin 4 [61,62]. However, this activity was statistically significant only for peptides obtained from spelt flour fermented with LAB, suggesting that specific peptides in this mixture are responsible for activating immune-related pathways in intestinal organoids. The increase in antimicrobial peptides further confirms their beneficial role, since the reduction of harmful intestinal bacteria is associated with decreased inflammatory responses.

The results obtained in this work therefore allow us to both confirm the antioxidant and anti-inflammatory activity of wheat flour peptides, as reported in the literature [25,37], and to extend these properties to peptides obtained from spelt flour. Intracellular ROS reduction represents a hallmark of bioactive peptides’ biological activity and it provides the basis for additional health-promoting effects, including anti-inflammatory activity. These two activities are, in fact, mutually dependent: some peptides present in the complex mixture used to treat the cells may activate antioxidant signaling pathways either by directly interacting with LPS or, once transported into the cells, by interacting with transcription factors that regulate anti-inflammatory pathways as reported for peptides from Jack bean protein hydrolysates [63]. The amino acid composition of the peptides can also determine their biological activity. The peptides are rich in aliphatic hydrophobic amino acids as well as in Gln and Pro whose antioxidant activities, due to a scavenging mechanism, are known [64]. Among the wide range of anti-inflammatory activities attributed to bioactive peptides [65], the ability of peptides from both wheat and spelt to restore intestinal function by upregulating Connexin 43 and lowering Cxcl1 is particularly noteworthy, as it directly involves the epithelial surface with which these peptides first interact and not previously described in literature. Moreover, the results suggest that sourdough fermentation is important in producing bioactive peptides when the bread is obtained with wheat flour. Conversely, the use of spelt flour enables the production of peptides equipped with higher antioxidant and anti-inflammatory activity following both bakery yeast and sourdough fermentation

## 5. Conclusions

In this study, we demonstrate that the biological activities of food-derived bioactive peptides can be enhanced by selecting alternative grain matrices, such as spelt flour, and by harnessing the proteolytic activity of lactic acid bacteria during sourdough fermentation. The overall results obtained highlight the ability of peptides to counteract the inflammatory state of the intestinal barrier, making them excellent candidates for maintaining the physiological function of the epithelium even in the presence of an inflammatory state. Moreover, all peptides were found to induce intestinal epithelial cells to both increase the expression of gap-junction proteins and reduce the level of pro-inflammatory cytokines, thereby making the epithelial membrane intact and resistant. Finally, it is of peculiar interest to observe that wheat flour samples exhibit higher antioxidant and anti-inflammatory activity when the respective doughs are produced using lactic acid fermentation. Meanwhile, samples from spelt flour doughs demonstrate comparable biological activity on cells, suggesting that spelt flour contains intrinsic protein sequences capable of producing bioactive peptides regardless of the leavening method used. This attests how the choice of both flour and fermentation method works synergistically to determine the nutritional and healthful qualities of the baked product.

## Figures and Tables

**Table 1:** Ingredients for bread-making.

	Breads			
Ingredients	W-Sac	W-LAB	S-Sac	S-LAB
Wheat flour type 00 (g)	525	525	--	--
Spelt flour (g)	--	--	525	525
Water (g)	225	225	225	225
Sourdough (g)	--	250	--	250
Baker’s yeast (g)	10	--	10	--
Dough yield (DY)	153	153	153	153

W-Sac: dough with wheat flour fermented by baker’s yeasts; W-LAB: dough with wheat flour fermented by a sourdough; S-Sac: dough with spelt flour fermented by baker’s yeasts; S-LAB: dough with spelt flour fermented by a sourdough.

**Table 2:** pH, total titratable acidity (TTA), volume increase (%, ∆V/V0), and LAB and yeast microorganism concentrations of the doughs at the end of the fermentation.

Sample	Final pH	Final TTA (mL)	ΔV/V × 100	LAB (CFU/g)	Yeasts (CFU/g)
W-Sac	5.76 ± 0.46 ^b^	3.64 ± 0.29 ^ab^	144.4 ± 10.1 ^a^	--	(1.35 ± 0.20) × 10^8 b^
W-LAB	4.21 ± 0.46 ^a^	5.34 ± 0.43 ^c^	138.9 ± 11.1 ^a^	(7.55 ± 0.20) × 10^8 a^	(3.11 ± 0.30) × 10^7 a^
S-Sac	5.85 ± 0.64 ^b^	2.77 ± 0.19 ^a^	144.4 ± 11.6 ^a^	--	(1.40 ± 0.88) × 10^8 b^
S-LAB	4.22 ± 0.34 ^a^	4.40 ± 0.48 ^b^	133.3 ± 9.3 ^a^	(7.99 ± 0.16) × 10^8 a^	(1.07 ± 0.07) × 10^7 a^

∆: variation between the final and the initial values. W-Sac: dough with wheat flour fermented by baker’s yeasts; W-LAB: dough with wheat flour fermented by a sourdough; S-Sac: dough with spelt flour fermented by baker’s yeasts; S-LAB: dough with spelt flour fermented by a sourdough. Results are expressed as average ± standard deviation. Values in the same column with different letters (a–b) are significantly different (*p* < 0.05).

**Table 3:** Protein content of each sample according to the Bradford assay.

Sample	Protein content (mg/mL)
W-Sac	0.89 ± 0.19 ^a^
W-LAB	1.77 ± 0.08 ^b^
S-Sac	1.15 ± 0.05 ^a^
S-LAB	1.78 ± 0.24 ^b^

Results are expressed as average ± standard deviation. Values in the same column with different letters (a–b) are significantly different (*p* < 0.05).

**Table 4:** Total free amino acid content in WSE extracts.

Sample	FAA content (mg/L)
W-Sac	241.1 ± 10.3 ^a^
W-LAB	443.1 ± 20.1 ^b^
S-Sac	444.5 ± 15.4 ^b^
S-LAB	411.1 ± 12.6 ^b^

Results are expressed as average ± standard deviation. Values in the same column with different letters (a–b) are significantly different (*p* < 0.05).

**Table 5:** Amino acid composition of bioactive peptides.

Amino Acid	W-Sac	W-LAB	S-Sac	S-LAB
Ala (A)	5.5%	8.8%	4.2%	4.9%
Arg (R)	2.9%	3.1%	3.2%	3.7%
Asn (N)	2.7%	2.8%	2.8%	2.9%
Asp (D)	1.6%	2.5%	1.2%	1.7%
Cys (C)	0.1%	0.2%	0.7%	1.2%
Gln (Q)	19.3%	10.8%	25.4%	21.7%
Glu (E)	3.2%	4.3%	3.0%	3.3.%
Gly (G)	9.1%	11.5%	6.0%	6.2%
His (H)	1.2%	1.6%	1.2%	1.5%
Ile (I)	2.8%	4.6%	3.4%	4.5%
Leu (L)	6.4%	8.0%	7.9%	9.2%
Lys (K)	1.9%	3.9%	1.9%	2.8%
Met (M)	0.9%	1.6%	1.8%	2.0%
Phe (F)	2.8%	2.8%	2.8%	2.5%
Pro (P)	13.8%	9.9%	14.4.%	12.3%
Ser (S)	6.4%	6.9%	6.0%	5.5%
Thr (T)	5.8%	5.0%	2.8%	3.1%
Trp (W)	0.3%	0.4%	0.4%	0.6%
Tyr (Y)	1.4%	1.4%	2.2%	2.1%
Val (V)	11.7%	10.1%	8.6%	8.2%

Amino acid composition of the peptides determined with the ProtParam tool at https://web.expasy.org/protparam/ (accessed on 3 September 2025)

## Data Availability

The original contributions presented in this study are included in the article/Supplementary Material. Further inquiries can be directed to the corresponding author.

## Supplementary Materials

The following supporting information can be downloaded at https://www.mdpi.com/article/10.3390/foods14183184/s1: Table S1: Primers used in detection of markers in jejunum organoids.; Table S2: Spelt proteins identified by mass spectrometry, Figure S1: Venn diagram and overlapping peptides identified by mass spectrometry.

## Abbreviations

The following abbreviations are used in this manuscript:

BPs	Bioactive peptides
LAB	Lactobacilli
WSE	Water-soluble extracts
W-Sac	Dough with wheat flour fermented by baker’s yeasts
W-LAB	Dough with wheat flour fermented by a sourdough
S-Sac	Dough with spelt flour fermented by baker’s yeasts
S-LAB	Dough with spelt flour fermented by a sourdough

## References

[1] Poole N., Donovan J., Erenstein O. (2022). Continuing Cereals Research for Sustainable Health and Well-Being. Int. J. Agric. Sustain..

[2] Shewry P.R., Hey S.J. (2015). The Contribution of Wheat to Human Diet and Health. Food Energy Secur..

[3] Marinangeli C.P.F., Nosworthy M.G., Shoveller A.-K. (2024). Cereal Proteins in the Human Diet: Reflecting on Their Contributions to Daily Protein Intake. J. Cereal Sci..

[4] Pequeno D.N.L., Ferreira T.B., Fernandes J.M.C., Singh P.K., Pavan W., Sonder K., Robertson R., Krupnik T.J., Erenstein O., Asseng S. (2024). Production Vulnerability to Wheat Blast Disease Under Climate Change. Nat. Clim. Change.

[5] Zhang T., He Y., DePauw R., Jin Z., Garvin D., Yue X., Anderson W., Li T., Dong X., Zhang T. (2022). Climate Change May Outpace Current Wheat Breeding Yield Improvements in North America. Nat. Commun..

[6] Ortiz A.M.D., Outhwaite C.L., Dalin C., Newbold T. (2021). A Review of the Interactions between Biodiversity, Agriculture, Climate Change, and International Trade: Research and Policy Priorities. One Earth.

[7] Sugár E., Fodor N., Sándor R., Bónis P., Vida G., Árendás T. (2019). Spelt Wheat: An Alternative for Sustainable Plant Production at Low N-Levels. Sustainability.

[8] Dumalasová V., Grausgruber H., Zelba O., Hanzalová A., Buerstmayr H., Weyermann V., dell’Avo F., Cuendet C., Koppel R., Sooväli P. (2025). Spelt Wheat Resistance to Rusts, Powdery Mildew, Leaf Blotch and Common Bunt. Cereal Res. Commun..

[9] Ruibal-Mendieta N.L., Delacroix D.L., Mignolet E., Pycke J.-M., Marques C., Rozenberg R., Petitjean G., Habib-Jiwan J.-L., Meurens M., Quetin-Leclercq J. (2005). Spelt (*Triticum aestivum* ssp. *spelta*) as a Source of Breadmaking Flours and Bran Naturally Enriched in Oleic Acid and Minerals but Not Phytic Acid. J. Agric. Food Chem..

[10] Frakolaki G., Giannou V., Topakas E., Tzia C. (2018). Chemical Characterization and Breadmaking Potential of Spelt Versus Wheat Flour. J. Cereal Sci..

[11] Geisslitz S., Wieser H., Scherf K.A., Koehler P. (2018). Gluten Protein Composition and Aggregation Properties as Predictors for Bread Volume of Common Wheat, Spelt, Durum Wheat, Emmer and Einkorn. J. Cereal Sci..

[12] Petrova P., Petrov K. (2020). Lactic Acid Fermentation of Cereals and Pseudocereals: Ancient Nutritional Biotechnologies with Modern Applications. Nutrients.

[13] Gabriele M., Arouna N., Árvay J., Longo V., Pucci L. (2023). Sourdough Fermentation Improves the Antioxidant, Antihypertensive, and Anti-Inflammatory Properties of Triticum Dicoccum. Int. J. Mol. Sci..

[14] Coda R., Cagno R.D., Gobbetti M., Rizzello C.G. (2014). Sourdough Lactic Acid Bacteria: Exploration of Non-Wheat Cereal-Based Fermentation. Food Microbiol..

[15] Alkay Z., Falah F., Cankurt H., Dertli E. (2024). Exploring the Nutritional Impact of Sourdough Fermentation: Its Mechanisms and Functional Potential. Foods.

[16] Gänzle M.G., Loponen J., Gobbetti M. (2008). Proteolysis in Sourdough Fermentations: Mechanisms and Potential for Improved Bread Quality. Trends Food Sci. Technol..

[17] Lu S., An M., Sun R., Luo H., Wen Y., Li H., Wang J., Sun B. (2025). Organic Acid Profiles of Traditional Sourdough Microbiota Dominates the Fermentation Effect on Whole Wheat Bread Texture. J. Cereal Sci..

[18] Wang Z., Zheng Y., Dai Y., Yang R., Zhao R., Sun G., Zhou W.-W., Feng S., Feng Y., Li N. (2024). Effect of Probiotic Fermentation on the Extraction Rate and Bioactivity of Plant-Based Polysaccharides: A Review. Innov. Food Sci. Emerg. Technol..

[19] Gobbetti M., Rizzello C.G., Di Cagno R., De Angelis M. (2014). How the Sourdough May Affect the Functional Features of Leavened Baked Goods. Food Microbiol..

[20] Chen L., Hui Y., Gao T., Shu G., Chen H. (2021). Function and Characterization of Novel Antioxidant Peptides by Fermentation with a Wild Lactobacillus Plantarum 60. LWT.

[21] Tonolo F., Fiorese F., Moretto L., Folda A., Scalcon V., Grinzato A., Ferro S., Arrigoni G., Bindoli A., Feller E. (2020). Identification of New Peptides from Fermented Milk Showing Antioxidant Properties: Mechanism of Action. Antioxidants.

[22] Bhandari D., Rafiq S., Gat Y., Gat P., Waghmare R., Kumar V. (2020). A Review on Bioactive Peptides: Physiological Functions, Bioavailability and Safety. Int. J. Pept. Res. Ther..

[23] Akbarian M., Khani A., Eghbalpour S., Uversky V.N. (2022). Bioactive Peptides: Synthesis, Sources, Applications, and Proposed Mechanisms of Action. Int. J. Mol. Sci..

[24] Ye H., Tao X., Zhang W., Chen Y., Yu Q., Xie J. (2022). Food-Derived Bioactive Peptides: Production, Biological Activities, Opportunities and Challenges. J. Future Foods.

[25] Galli V., Mazzoli L., Luti S., Venturi M., Guerrini S., Paoli P., Vincenzini M., Granchi L., Pazzagli L. (2018). Effect of Selected Strains of Lactobacilli on the Antioxidant and Anti-Inflammatory Properties of Sourdough. Int. J. Food Microbiol..

[26] Lin H., Zhao J., Xie Y., Tang J., Wang Q., Zhao J., Xu M., Liu P. (2024). Corrigendum to “Identification and Molecular Mechanisms of Novel Antioxidant Peptides from Fermented Broad Bean Paste: A Combined In Silico and In Vitro Study” [Food Chemistry 450 (2024) 139297/FOCH_FOODCHEM-D-23-08808]. Food Chem..

[27] Tyagi A., Chelliah R., Daliri E.B.-M., Sultan G., Madar I.H., Kim N.-H., Shabbir U., Oh D.-H. (2023). Antioxidant Activities of Novel Peptides from Limosilactobacillus Reuteri Fermented Brown Rice: A Combined In Vitro and In Silico Study. Food Chem..

[28] Li Y., Gao X., Pan D., Liu Z., Xiao C., Xiong Y., Du L., Cai Z., Lu W., Dang Y. (2023). Identification and Virtual Screening of Novel Anti-Inflammatory Peptides from Broccoli Fermented by Lactobacillus Strains. Front. Nutr..

[29] Xian Y., Da P., Chao Y., Hui X., Ligang Y., Shaokang W., Guiju S. (2022). Wheat Oligopeptides Enhance the Intestinal Mucosal Barrier and Alleviate Inflammation via the TLR4/Myd88/MAPK Signaling Pathway in Aged Mice. Food Nutr. Res..

[30] Cicchi C., Paoli P., Modesti A., Mannelli F., Scicutella F., Buccioni A., Fontanarosa C., Luti S., Pazzagli L. (2023). Effect of Bovine Milk Peptides on Cell Inflammation, Proliferation and Differentiation: Milk Potential Benefits Are Preserved in an Unconventional Cow Feeding Strategy. Biology.

[31] González-Montoya M., Hernández-Ledesma B., Silván J.M., Mora-Escobedo R., Martínez-Villaluenga C. (2018). Peptides Derived from in Vitro Gastrointestinal Digestion of Germinated Soybean Proteins Inhibit Human Colon Cancer Cells Proliferation and Inflammation. Food Chem..

[32] Jing Y., Liu X., Wang J., Zheng X. (2024). Corn Protein Hydrolysate with Glutamine-Rich Peptides Protects Intestinal Barrier in Caco-2 Cells: Insights into Structural Characteristics of Identified Glutamine Peptides. J. Funct. Foods.

[33] Liang Q., Ren X., Chalamaiah M., Ma H. (2020). Simulated Gastrointestinal Digests of Corn Protein Hydrolysate Alleviate Inflammation in Caco-2 Cells and a Mouse Model of Colitis. J. Food Sci. Technol..

[34] Ji Z.-H., Xie W.-Y., Zhao P.-S., Wu H.-Y., Ren W.-Z., Hu J.-P., Gao W., Yuan B. (2023). Oat Peptides Alleviate Dextran Sulfate Sodium Salt-Induced Colitis by Maintaining the Intestinal Barrier and Modulating the Keap1-Nrf2 Axis. Nutrients.

[35] Jing Y., Liu X., Wang J., Ma Y., Zheng X. (2022). Production of Corn Protein Hydrolysate with Glutamine-Rich Peptides and Its Antagonistic Function in Ulcerative Colitis In Vivo. Foods.

[36] Zhang B., Xu Y., Zhao C., Zhang Y., Lv H., Ji X., Wang J., Pang W., Wang X., Wang S. (2022). Protective Effects of Bioactive Peptides in Foxtail Millet Protein Hydrolysates against Experimental Colitis in Mice. Food Funct..

[37] Luti S., Mazzoli L., Ramazzotti M., Galli V., Venturi M., Marino G., Lehmann M., Guerrini S., Granchi L., Paoli P. (2020). Antioxidant and Anti-Inflammatory Properties of Sourdoughs Containing Selected Lactobacilli Strains Are Retained in Breads. Food Chem..

[38] Graça C., Lima A., Raymundo A., Sousa I. (2021). Sourdough Fermentation as a Tool to Improve the Nutritional and Health-Promoting Properties of Its Derived-Products. Fermentation.

[39] D’Amico V., Gänzle M., Call L., Zwirzitz B., Grausgruber H., D’Amico S., Brouns F. (2023). Does Sourdough Bread Provide Clinically Relevant Health Benefits?. Front. Nutr..

[40] Kelly M.T., Blaise A., Larroque M. (2010). Rapid Automated High Performance Liquid Chromatography Method for Simultaneous Determination of Amino Acids and Biogenic Amines in Wine, Fruit and Honey. J. Chromatogr. A.

[41] Smith P.K., Krohn R.I., Hermanson G.T., Mallia A.K., Gartner F.H., Provenzano M.D., Fujimoto E.K., Goeke N.M., Olson B.J., Klenk D.C. (1985). Measurement of Protein Using Bicinchoninic Acid. Anal. Biochem..

[42] Bradford M.M. (1976). A Rapid and Sensitive Method for the Quantitation of Microgram Quantities of Protein Utilizing the Principle of Protein-Dye Binding. Anal. Biochem..

[43] Taciak B., Białasek M., Braniewska A., Sas Z., Sawicka P., Kiraga Ł., Rygiel T., Król M. (2018). Evaluation of Phenotypic and Functional Stability of RAW 264.7 Cell Line through Serial Passages. PLoS ONE.

[44] More G.K., Makola R.T. (2020). In-Vitro Analysis of Free Radical Scavenging Activities and Suppression of LPS-Induced ROS Production in Macrophage Cells by Solanum Sisymbriifolium Extracts. Sci. Rep..

[45] Ding X., Hu X., Chen Y., Xie J., Ying M., Wang Y., Yu Q. (2021). Differentiated Caco-2 Cell Models in Food-Intestine Interaction Study: Current Applications and Future Trends. Trends Food Sci. Technol..

[46] Panse N., Gerk P.M. (2022). The Caco-2 Model: Modifications and Enhancements to Improve Efficiency and Predictive Performance. Int. J. Pharm..

[47] Córdova S., Tena-Garitaonaindia M., Álvarez-Mercado A.I., Gámez-Belmonte R., Gómez-Llorente M.A., de Medina F.S., Martínez-Cañavate A., Martínez-Augustin O., Gómez-Llorente C. (2024). Differential Modulation of Mouse Intestinal Organoids with Fecal Luminal Factors from Obese, Allergic, Asthmatic Children. Int. J. Mol. Sci..

[48] Minervini F., Lattanzi A., De Angelis M., Di Cagno R., Gobbetti M. (2012). Influence of Artisan Bakery- or Laboratory-Propagated Sourdoughs on the Diversity of Lactic Acid Bacterium and Yeast Microbiotas. Appl. Environ. Microbiol..

[49] Oliveros J.C. (2007–2015) Venny. An Interactive Tool for Comparing Lists with Venn’s Diagrams. https://bioinfogp.cnb.csic.es/tools/venny/index.html.

[50] Chatonidi G., Pradal I., De Vuyst L., Courtin C.M., Verbeke K. (2025). Effect of Lactic Acid-Rich Sourdough Bread on Appetite Regulation: A Randomized, Double-Blind Controlled Trial. Curr. Res. Food Sci..

[51] Pérez-Vega K.A., Sanllorente A., Zomeño M.-D., Quindós A., Muñoz-Martínez J., Malcampo M., Aldea-Perona A., Hernáez Á., Lluansí A., Llirós M. (2024). Sourdough Bread with Different Fermentation Times: A Randomized Clinical Trial in Subjects with Metabolic Syndrome. Nutrients.

[52] Geisslitz S., Scherf K.A. (2020). Rediscovering Ancient Wheats. Cereal Foods World.

[53] Shewry P. (2023). Wheat Grain Proteins: Past, Present, and Future. Cereal Chem..

[54] Paramithiotis S., Gioulatos S., Tsakalidou E., Kalantzopoulos G. (2006). Interactions between Saccharomyces Cerevisiae and Lactic Acid Bacteria in Sourdough. Process Biochem..

[55] Tóth V., Láng L., Vida G., Mikó P., Rakszegi M. (2022). Characterization of the Protein and Carbohydrate Related Quality Traits of a Large Set of Spelt Wheat Genotypes. Foods.

[56] Diana M., Rafecas M., Quílez J. (2014). Free Amino Acids, Acrylamide and Biogenic Amines in Gamma-Aminobutyric Acid Enriched Sourdough and Commercial Breads. J. Cereal Sci..

[57] Dogan C.E., Cebi N., Develioglu A., Olgun E.O., Sagdic O. (2018). Detection of Cystine and Cysteine in Wheat Flour Using a Robust LC-MS/MS Method. J. Cereal Sci..

[58] Zhu Y., Lao F., Pan X., Wu J. (2022). Food Protein-Derived Antioxidant Peptides: Molecular Mechanism, Stability and Bioavailability. Biomolecules.

[59] Martínez-Augustin O., Rivero-Gutiérrez B., Mascaraque C., de Medina F.S. (2014). Food Derived Bioactive Peptides and Intestinal Barrier Function. Int. J. Mol. Sci..

[60] Korbecki J., Maruszewska A., Bosiacki M., Chlubek D., Baranowska-Bosiacka I. (2022). The Potential Importance of CXCL1 in the Physiological State and in Noncancer Diseases of the Cardiovascular System, Respiratory System and Skin. Int. J. Mol. Sci..

[61] Ganz T. (2003). Defensins: Antimicrobial Peptides of Innate Immunity. Nat. Rev. Immunol..

[62] Sultana M.F., Suzuki M., Yamasaki F., Kubota W., Takahashi K., Abo H., Kawashima H. (2022). Identification of Crucial Amino Acid Residues for Antimicrobial Activity of Angiogenin 4 and Its Modulation of Gut Microbiota in Mice. Front. Microbiol..

[63] Wijatniko B.D., Yamamoto Y., Hirayama M., Suzuki T. (2024). Identification and Molecular Mechanism of Anti-Inflammatory Peptides Isolated from Jack Bean Protein Hydrolysates: In Vitro Studies with Human Intestinal Caco-2BBe Cells. Plant Foods Hum. Nutr..

[64] Zhou N., Zhong Y., Liu H. (2024). Characterization and Relationship Analysis of Antioxidant and Anti-Inflammatory Peptides in Pomelo Fruitlet Albumin. Food Chem..

[65] Guha S., Majumder K. (2019). Structural-Features of Food-Derived Bioactive Peptides with Anti-Inflammatory Activity: A Brief Review. J. Food Biochem..

