# Supplementary material for: Sourdough Breads Made with Selected Lactobacillus Strains and Spelt Flour Contain Peptides That Positively Impact Intestinal Barrier
Source: Foods. 2025 Sep 12;14(18):3184. doi: 10.3390/foods14183184 (PMC12470101; doi:10.3390/foods14183184)
Supplement: Supplementary file 1 [file foods-14-03184-s001.zip › foods-3831622-supplementary.pdf]

A) Venn diagram

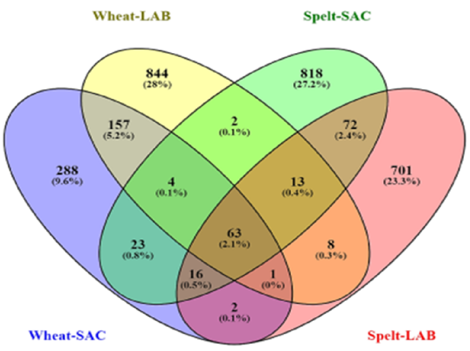

D) 8 unique peptides in Wheat-LAB e Spelt-LAB

PQAQGSVQPQQLPQF
VQGQGIIQPQQPA
VQGQGIIQPQQPAQ
VQGQGIIQPQQPAQL
LVQGQGIIQPQQPAQL
VQGQGIIQPQQPAQLE
IALPVPSQPVD
VVRQYEQTVVPPKGGSFYPGETTPLQQL

Figure S1: A) Venn diagram obtained with the peptide sequences from mass spectrometry . Diagram was obtined at at https://bioinfogp.cnb.csic.es/tools/venny/index.html .
B) Peptides in common with all samples. The sequences come predominantly from wheat and spelt prolamins . **C)** Unique peptides of spelt flour regardless of leavening method. The sequences belong to alpha-gliadin and prolamin families. **D)** Unique peptides from wheat and spelt breads obtained with sourdoughs. The sequences belong to alpha and gamma-gliadin and to HMW glutenin.
Wheat-Sac: dough with wheat flour fermented by baker's yeasts; Wheat-LAB: dough with wheat flour fermented by sourdough; Spelt-Sac: dough with spelt flour fermented by baker's yeasts; Spelt-LAB: dough with spelt flour fermented by sourdough.

B) 63 peptides common to all samples

VRVPVPQL
VRVPVPQLQ
VRVPVPQLQPQN
VRVPVPQLQPQNP
VRVPVPQLQPQNPS
VRVPVPQLQPQNPSQ
VRVPVPQLQPQNPSQQ
VPVPQLQPQNPSQQQPQ
VRVPVPQLQPQNPSQQQ
VRVPVPQLQPQNPSQQQP
VPVPQLQPQNPSQQQPQE
VRVPVPQLQPQNPSQQQPQ
VRVPVPQLQPQNPSQQQPQE
VRVPVPQLQPQNPSQQQPQEQ
VRVPVPQLQPQNPSQQQPQEQV
VRVPVPQLQPQNPSQQQPQEQVP
VRVPVPQLQPQNPSQQQPQEQVPL
VRVPVPQLQPQNPSQQQPQEQVPLV
VRVPVPQLQPQNPSQQQPQEQVPLVQ
VRVPVPQLQPQNPSQQQPQEQVPLVQQ
VRVPVPQLQPQNPSQQQPQEQVPLVQQQ
VRVPVPQLQPQNPSQQQPQEQVPLVQQQQ
VRVPVPQLQPQNPSQQQPQEQVPLVQQQQF
VRVPVPQLQPQNPSQQQPQEQVPLVQQQQFPG
VRVPVPQLQPQNPSQQQPQEQVPLVQQQQFPGQ
VRVPVPQLQPQNPSQQQPQEQVPLVQQQQFPGQQ
VRVPVPQLQPQNPSQQQPQEQVPLVQQQQFPGQQQ
VRVPVPQLQPQNPSQQQPQEQVPLVQQQQFL
VRVPVPQLQPQNPSQQQPQEQVPLVQQQQFLG
VRVPVPQLQPQNPSQQQPQEQVPLVQQQQFLGQQ
VRVPVPQLQPQNPSQQQPQEQVPLMQQQ
VRVPVPQLQPQNPSQQQPQEQVPLVQE
VRVPVPQLQPQNPSQQQPQEQVPLVQEQ
VRVPVPQLQPQNPSQQQPQEQVPLVQEQQ
VRVPVPQLQPQNPSQQQPQEQVPLVQEQQF
STTIAPFGIFGTN
VRVPVPQLQPQNPSQQQPQKQVPLVQQ
VRVPVPQLQPQNPSQQQPQKQVPLVQQQ
VRVPVPQLQPQNPSQQQPQKQVPLVQQQQ
VRVPVPQLQPKNPSQQQPQEQVPLVQQ
VRVPVPQLQPKNPSQQQPQEQVPLVQQQ
VRVPVPQLQPKNPSQQQPQEQVPLVQQQQ
VRVPVPQLQPKNPSQQQPQEQVPLVQQQQF
VRVPVPQLQPKNPSQQQPQEQVPLVQQQQFPG
VRVPVPQLQPKNPSQQQPQEQVPLVQQQQFPGQ
VRVPVPQLQPKNPSQQQPQEQVPLVQQQQFPGQQ
VRVPVPQLQPKNPSQQQPQEQVPLVQQQQFPGQQQ
VRVPVPQLQPQNPSQQQSQEQVP
VRVPVPQLQPQNPSQQQSQEQVPL
VRVPVPQLQPQNPSQQQSQEQVPLVQQ
VRVPVPQLQPQNPSQQQSQEQVPLVQQQ
VRVPVPQLQLQNPSQQQPQEQVPL
VRVPVPQLQLQNPSQQQPQEQVPLVQ
VRVPVPQLQLQNPSQQQPQEQVPLVQE
VRFPVPQLQPQNPSQQQPQEQVPL
VRFPVPQLQPQNPSQQQPQEQVPLVQQ
VRFPVPQLQPQNPSQQQPQEQVPLVQQQQ
VRVPVPQLQPQNPSQQQPQEQVPLVQQQFLGQ
VRVPVPQLQPQNPSQQQPQEQVPLVQQQFLGQQQ
VRVPVPQLEPQN
PFSSVVAGIGGQ
SIIKAPFSSVVAGIGGQ
VYYPTSPQQPGQLQQPAQGQQ

C) 72 unique peptides in Spelt-SAC and Spelt-LAB

VRVPVP
APFGIFGTN
TAVRVPVPQLQPQNP
VRVPVPQLQPQNPSQQQPQEQVPLVQQQQFLGQQQPF
VRVPVPQLQPQNPSQQQPQEQVPLVQQQQFLGQQQPFPPQQPYPQPQPFPSQQP
PYPQPQPFRPQQPY
TTTAVRVPVPQLQPQNPSQQQPQEQVPLVQ
VRVPVPQLQPQNPSQQQPQEQVPLVQQQQFLGQQQQQFPGQ
IVATTATI
QAIHNVVHAIILHQQHHHHQQQ
FQQPQQQYPSGQGFFQPSQQNPQAQGSFQPQQLPQFEAIRNLALQTLP
QQLIPCMYVVLQQHNIAQGRSQV
YLQLQPFPQPQLPYSQPQPFQPQQP
AVRVPVPQLQPQNPSQQQPQEQVPVVQQQQFL
VPVPQLQPQNPSQQQPQKQVPLVQQQQ
VRVPVPQLQPQNPSQQQPQKQVPLVQQQQFP
LALQMLPAMCNVYIPPHCS
VRVPVPQLQPQNPSQQQSQEQVPLVQQQQ
VRVPVPQLQPKNPSQQQPQEQVP
PQLQLQNP
VRVPVPQLQLQNPS
VRVPVPQLQLQNPSQQQPQEQVPLVQEQQ
VRFPVPQLQPQNPSQQQPQ
VRFPVPQLQPQNPSQQQPQEQVPLVQ
QKQQQELSSQ
VRVSVPQLQPQNPSQQQPQEQVPL
VRVSVPQLQPQNPSQQQPQEQVPLVQ
VRVSVPQLQPQNPSQQQPQEQVPLVQQQQF
VQQLQPQNPSQQQPQEQVPLV
TTAVRVPVQQLQPQNPSQQQPQEQVPLV
PYLQLQPFLQP
LQLQPFPQPQP
LGQQQQFPPQQ
DVVLQQHNIAHGSSQVLQQSTYQL
QQQYPSGQGSFRPSQQNPQAQGSVQPQQ
QLWKIPEQSRCQAIHNVVHAIILHQQQQ
AVRVPVPQLQPQNPSQQQPQGQVPLVQQQQFLGQQ
AVRVPVPQLQPQNPSQQQPQGQVPLVQQQQFLGQQQP
VRVPVPQLQLQNPSQQQPQEQVPLVQQQQFP
VRVPVPQLQLQNPSQQQPQEQVPLVQQQQFPG
VRVPVPQLQLQNPSQQQPQEQVPLVQQQQFPGQQ
PQEHVPLVQQ
PYLQLQPFPS
PLPQPQPFLPQLP
RCQAIQNVVHA
VRVPVPQLQSQNPSQQQPQEQVP
VRVPVPQLQSQNPSQQQPQEQVPL
RAPVPQLQPQNPSQQQP
LPLYQQQQV
FIQPSLQQQV
VQGQGIIQPQQP
IPQQLQCAAIHT
PTSPQQPGQGQQPG
LTSPQQS
TSPLQLGQG
RLEGGDALSASQ
IVVPPKGGSFYPGETTPPQQLQQ
AEQQAASPMVAKAQHPVTQLPTV
PGYHPTSSQLQLGQL
QGQQEYYLT
PTSQQQPGQGPQPG
VVVPPKGGSFYPGETTPPQ
VVVPPKGGSFYPGETTPPQQ
QPVGGGPVARQYEQQVVVPPKG
VVVPPKGGSFYPGETTPPQQLQQS
EYQAARLKVAKAQ
QPGQGQQPGQGQQSG
PITVSPGTRQYEQQPVVPS
QQVVDQQLRDVSPGCRPITV
PEQGQRPGQGQQGYYPTSPQQPG
SPYHVSAEYQAARLKVAKAQQLAAQLPA
QQGYYPTSLQQLGQGQQPGQGQPGYYPT

Figure S1: Venn diagram and overlapping peptides identified by mass spectrometry

Table S1: Primers used in RT-qPCR for determination of inflammatory and immune activation markers in mouse jejunum organoids

Gene	**Forward**	**Reverse**
18s	ACACGGACAGGATTGACAGATTG	GCCAGAGTCTCGTTCGTTATCG
Ang4	TTTGGAATCACTGTTGGAAG	TGCTGACGTAGGAATTTTTC
Cxcl1	CCGAAGTCATAGCCACACTCAAG	ACCAGACAGGTGCCAATCAGAG
Defa1	GTCCTACTCCTTTGCCCTTG	GCAGCCTCTTGATCTACAATA
Hprt	AGGGATTTGAATCACGTTTG	TTTACTGGCAACATCAACAG
Pyib	TGGAAGATGAATCTGTAGGAC	CAAATCCTTTCTCTCCTGTAG

Table S2: Spelt proteins identified by mass spectrometry.

Spelt - Sac					Spelt - LAB				
Family	*Member*	*Accession*	*Score*	*#sequenc.*	*Family*	*Member*	*Accession*	*Score*	*#sequenc.*
1	1	A0A1P8DSI8_9POAL - Prolamin	5022	87	1	1	A0A1P8DT72_9POAL- Prolamin	2833	59
1	2	A0A1P8DT94_9POAL	5006	87	1	2	A0A1P8DT37_9POAL	2659	58
1	3	A0A1P8DSP4_9POAL	4991	79	1	3	A0A0E3Z663_9POAL	2352	54
1	4	A0A1P8DSK6_9POAL	4842	74	1	4	A0A1P8DSM7_9POAL	2101	58
1	5	A0A1P8DSZ1_9POAL	4467	75	1	5	A0A1P8DSS5_9POAL	1861	50
1	6	A0A1P8DSS5_9POAL	4089	66	1	6	A0A1P8DTB3_9POAL	1385	53
1	7	A0A1P8DSM7_9POAL	3651	63	1	7	A0A0E3Z6W2_9POAL	1002	46
1	8	A0A0E3URD0_9POAL	3203	64	1	8	A0A1P8DTB4_9POAL	918	42
1	9	A0A1P8DTB3_9POAL	2705	51	1	9	A0A1P8DSQ5_9POAL	415	40
1	10	A0A0E3Z5E3_9POAL	1922	53	1	10	A0A1P8DSB1_9POAL	328	30
1	11	A0A1P8DTB4_9POAL	1817	56	1	11	A0A1P8DT35_9POAL	310	32
1	12	A0A1P8DSQ5_9POAL	1467	44	1	12	A0A1P8DT16_9POAL	160	30
1	13	A0A1P8DSP5_9POAL	1274	43	1	13	A0A1P8DSH9_9POAL	147	31
1	14	A0A1P8DT66_9POAL	960	56	2	1	Q9XEW0_9POAL gamma-gliadina	261	27
1	15	A0A1P8DSH9_9POAL	731	43	3	1	A0A7H1K1V6_9POAL inibitore a-amilasi	83	15
1	16	A0A1P8DT16_9POAL	723	43	4	1	S4U5H5_9POAL- HMW-glutenin sub D	54	37
1	17	A0A1P8DSI0_9POAL	492	48	4	2	Q7XZI2_9POAL HMW-glutenin sub-Y-	50	9
1	18	A0A1P8DSY4_9POAL	410	46	4	3	A0A2D2CI59_9POAL HMW-glutenin Y	39	33
1	19	A0A1P8DT27_9POAL	207	29	5	1	A0A5B9C2V1_9POAL - HME glutenin x type	29	50
2	1	Q9XEW0_9POAL Gamma-gliadin	332	53	6	1	A0A811BQP2_9POAL - Large ribosomal subunit protein	26	15
3	1	Q7XZI2_9POAL Y-type HMW glutenin subunit	102	13	7	1	A0A811BNJ3_9POAL Photosystem II reaction center protein H	23	7
3	2	A0A4Y5P868_9POAL HMW glutenin y-type subunit	85	28	8	1	A0A811BLF1_9POAL NADPH oxidoreductase subunit H	22	35
3	3	S4U1S9_9POAL - HMW glutenin subunit 1Dx	32	36	9	1	A0A811BLC4_9POAL ATP-dependent Clp protease	21	16
4	1	A0A5B9C2V1_9POAL HMW glutenin x-type subunit	41	42	10	1	A0A7H1K1U8_9POAL- alpha-amylase inhibitor	20	11
5	1	A0A5B9BZN2_9POAL HMW-glutenin x-type subunit 1Bx6.1	38	44	11	1	U3PLU9_9POAL- Truncated RGA-7B	18	22
6	1	A0A0K0QPS2_9POAL Cold-responsive protein WCOR15-2D	23	9	12	1	D7PDA3_9POAL - Tsn1 defence response	17	124
7	1	Q66NH1_9POAL Phytochrome	20	74	13	1	A0A811BMG4_9POAL RNA polymerase subunit beta	16	43
8	1	A0A1Z1D7U6_9POAL NAD(P)H-quinone oxidoreductase	18	39	14	1	A0A7D5MEE0_9POAL alpha-amylase inhibitor	16	13
9	1	A0A811BMS7_9POAL RNA polymerase subunit beta	16	65	15	1	A0A811BMH8_9POAL Photosystem I assembly protein	15	13
10	1	A0A811BP01_9POAL Small ribosomal subunit protein uS12c	16	6	16	1	A0A811BNW0_9POAL Small ribosomal subunit protein	15	25
11	1	A0A811BPF9_9POAL NAD(P)H-quinone oxidoreductase	15	18	17	1	A0A5B9BZN2_9POAL HMW glutenin x-type	14	29
12	1	A0A2H4KDM8_9POAL Ubiquinol oxidase	14	19	18	1	A0A811BL06_9POAL- Photosystem II D2 protein	14	23
13	1	A0A1B2RX53_9POAL ABCG-D transporter	14	14					
14	1	D0Q0D0_9POAL 15-cis-phytoene synthase	14	26					
15	1	H9AXN6_9POAL Starch synthase	14	52					

The acquired data were analyzed using the Mascot 2.4 search engine (Matrix Science Ltd., London, UK) against a Triticum and a Spelta databases created from NCBI.
Full scan from 300 to 1600 m/z in the Orbitrap analyzer, using a 1 x 106 target value. Other details are reported in the method section
